# Clinical characteristics and gene mutation profiles of chronic obstructive pulmonary disease in non-small cell lung cancer

**DOI:** 10.3389/fonc.2022.946881

**Published:** 2022-10-04

**Authors:** Lin Yuan, Ting Guo, Chengping Hu, Wei Yang, Xiaoli Tang, Hao Cheng, Yang Xiang, Xiangping Qu, Huijun Liu, Xiaoqun Qin, Ling Qin, Chi Liu

**Affiliations:** ^1^ Department of Respiratory Medicine, National Key Clinical Specialty, Branch of National Clinical Research Center for Respiratory Disease, Xiangya Hospital, Central South University, Changsha, China; ^2^ Department of Physiology, School of Basic Medicine Science, Central South University, Changsha, China; ^3^ Basic and Clinical Research Laboratory of Major Respiratory Diseases, Central South University, Changsha, China; ^4^ Department of Respiratory Medicine, The First Hospital of Changsha, Changsha, China; ^5^ National Clinical Research Center for Geriatric Disorders, Xiangya Hospital, Changsha, China; ^6^ Pulmonary and Critical Care Medicine, Huaihua Tumor Hospital, Huaihua, China; ^7^ Department of Radiotherapy, The Second Affiliated Hospital, Hengyang Medical School, University of South China, Hengyang, China; ^8^ Department of Nasopharyngeal Carcinoma, The First People’s Hospital of Chenzhou, Chenzhou, China

**Keywords:** chronic obstructive pulmonary disease, non-small cell lung cancer, NSCLC-related genes, tumor DNA, circulating tumor DNA

## Abstract

**Purpose:**

The coexistence of chronic obstructive pulmonary disease (COPD) often leads to a worse prognosis in patients with non-small cell lung cancer (NSCLC). Meanwhile, approaches targeting specific genetic alterations have been shown to significantly improve the diagnosis and treatment outcomes of patients with NSCLC. Herein, we sought to evaluate the impact of COPD on the clinical manifestations and gene mutation profiles of NSCLC patients with both circulating tumor (ctDNA) and tumor DNA (tDNA).

**Materials and methods:**

The influence of COPD on clinical features was observed in 285 NSCLC cohorts suffering from NSCLC alone, NSCLC coexisting with COPD, or NSCLC coexisting with prodromal changes in COPD (with emphysema, bullae, or chronic bronchitis). The gene mutation profiles of specific 168 NSCLC-related genes were further analyzed in the NSCLC sub-cohorts with formalin-fixed and paraffin-embedded tumor DNA (FFPE tDNA) samples and plasma circulating tumor DNA (PLA ctDNA) samples. Moreover, mutation concordance was assessed in tDNA and paired ctDNA of 110 NSCLC patients.

**Results:**

Relative to patients with NSCLC alone, patients with NSCLC coexisting with COPD and prodromal changes presented with worse lung functions, more clinical symptoms, signs and comorbidities, and inconsistent gene mutation profiles. In addition, patients in the latter two groups exhibited a higher average frequency of gene mutation. Lastly, mutation concordance between tDNA and ctDNA samples was significantly reduced in NSCLC patients coexisting with COPD.

**Conclusions:**

Collectively, our findings revealed that coexistence of COPD leads to worse clinical manifestations and altered gene mutation profiles in patients with NSCLC. Additionally, for NSCLC patients with COPD, the use of ctDNA instead of tDNA may not be the most efficient approach to identifying gene mutations.

## Introduction

Lung cancer (LC) and chronic obstructive pulmonary disease (COPD) are one of the leading causes of mortality across the world (1, 2). A plethora of evidence further indicates that COPD serves as the major and independent risk factor for LC (3–5). It is also noteworthy that COPD patients are at a higher risk of developing LC than those without COPD (6–8). Additionally, LC patients suffering from COPD are more susceptible to poor outcomes, such as decreased lung function, increased symptom severity, and shorter survival (9–11). Although smoking is a well-established major risk factor for the development of LC and COPD, another approach also suggests the involvement of genetic susceptibility in the course of these two diseases (3, 12). Gene mutations, such as single-nucleotide variants (SNVs) and copy number variants (CNVs), are further capable of modifying LC prevalence and prognosis by altering gene regulation and expression (13, 14).

One particular type of lung malignancy, namely non-small cell lung cancer (NSCLC), accounts for approximately 85% of all cases of LC (15). Unsurprisingly, recent evidence underscores the presence of different gene mutations in NSCLC (16). Meanwhile, a prior study further indicated the importance of gene mutations for the subclassification of NSCLC, which has paved the way for remarkable progress in the targeted therapies (16, 17). However, the difficulty of obtaining lung tumor biopsies poses a challenge for a number of NSCLC patients in many clinical situations. On the other hand, circulating tumor DNA (ctDNA) can be found in the peripheral blood of both early- and late-stage cancer patients (18). In addition, ctDNA has been previously shown to closely match tumor DNA (tDNA) (19–21), and further validated as a surrogate means for detecting mutations in NSCLC (22, 23). However, it is imperative to enhance our understanding of the impact of COPD on gene mutations of 168 NSCLC-related genes, and the consistency of gene mutations between tDNA and paired ctDNA in NSCLC patients.

As COPD largely develops from chronic bronchitis and emphysema (24), and pulmonary bulla is a common accompaniment of COPD (25), we speculated whether comorbid COPD or prodromal changes in COPD could influence the clinical characteristics and gene mutation profiles of NSCLC patients. Accordingly, the primary purpose of this study was to explore the impact of COPD on the clinical characteristics, gene mutation profiles of 168 NSCLC-related genes, and mutation consistency in tDNA and paired ctDNA of NSCLC patients. Additionally, the current study intends to investigate the impact of these three prodromal changes in COPD on these aspects of NSCLC patients.

## Materials and methods

### Study design

**Figure 1:**
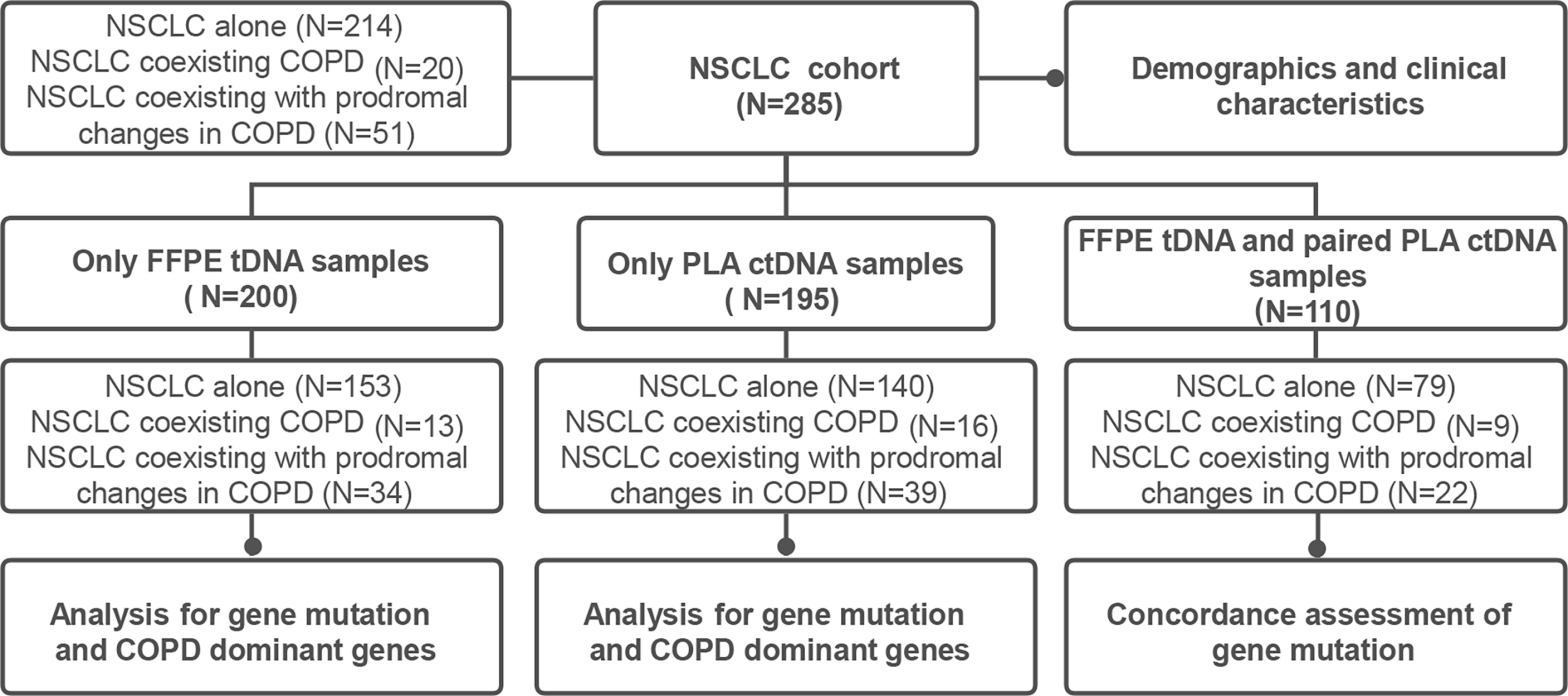
Overview of study design and sample size in our cohorts.

The current study was designed as a retrospective analysis for NSCLC patients, with the primary objective being the evaluation of the effect of COPD or its prodromal diseases on the clinical manifestations and gene mutation profiles of NSCLC patients. An additional goal included the investigation of the effects of COPD or its prodromal diseases on the consistency of tDNA and ctDNA from NSCLC patients (**Figure 1**). The study was approved by the Xiangya Hospital Ethics Review Committee (No. 202204086). All samples and related data in this study were irreversibly anonymized.

### Patient selection and sample sources

This study enrolled a total of 285 patients with NSCLC at Xiangya Hospital of Central South University (Changsha, Hunan, China) between February 2016 and May 2019. The inclusion criteria for the enrolled patients were as follows: (1) patients were diagnosed with NSCLC by two or more pathologists from Xiangya Hospital; (2) patients had not undergone systemic therapies, including chemotherapy, radiotherapy, targeted therapy, immunotherapy, etc. before enrollment; (3) patients can provide matching tissue samples or plasma samples; (4) patients presented with complete clinical data and pathological data. The patients not meeting the aforementioned inclusion criteria were excluded from the study. Additional exclusion criteria included the coexistence with other types of cancers. Thereafter, formalin-fixed and paraffin-embedded (FFPE) and plasma (PLA) samples were collected at baseline from the included patients prior to treatment.

### Data collection

The included 285 NSCLC patients were divided into the following three groups: NSCLC alone, NSCLC coexisting with COPD (previously diagnosed as COPD) and NSCLC coexisting with prodromal changes in COPD (with history or CT with emphysema, bullae, or chronic bronchitis). A range of data were collected which included the age, sex, smoking status, lung function, cell type, UICC stage, family history of NSCLC, presenting symptoms and signs, and comorbidity. Body mass index (BMI) was calculated as the weight in kilograms and divided by height in meters squared. The pulmonary function test was performed in accordance with the previously published literature (26). Among the 285 participants, there were 31 patients in the NSCLC alone group, 3 patients in the NSCLC coexisting COPD group, and 7 patients in the NSCLC coexisting with prodromal changes in COPD group lacking UICC stage information.

### Sample DNA preparation, next generation sequencing (NGS) library preparation and DNA sequencing

Total DNA content from tumor tissue and blood samples was processed in accordance with the previously published literature (27). For library preparation of 168 NSCLC-related genes from a commercial gene panel, fragments of 200 - 400 bp were chosen by the use of AMPure beads (Agencourt AMPure XP Kit), and then hybridized with the capture probe baits, used the magnetic beads for hybridization selection and amplified by PCR. The indexed samples were sequenced on a Nextseq500 sequencer (Illumina, Inc., USA) with paired-end reads. An average depth exceeded 10,000 ×(21).

### Statistical analysis

Quantitative data were expressed as mean ± standard deviation (SD). Qualitative data were described using relative frequencies. Characteristic data of recruited NSCLC patients were analyzed using Chi-squared test, Fisher exact test or Mann-Whitney U test. For the analysis of gene mutation profiles, (1) the positive rate of gene mutation was defined as the proportion of samples with gene mutations in the total number of samples; (2) the maxAF was defined as the maximum allelic fraction of all somatic mutations identified in the sample; and (3) FFPE tDNA was adopted as the previous reference when compared to PLA ctDNA. Matched FFPE tDNA and PLA ctDNA samples with the same mutations were regarded as true positives. Matched sample pairs without mutations (wild type) in the 168 NSCLC-related genes were regarded as true negatives. Besides, the sample pairs with mutations identified in PLA ctDNA but not FFPE tDNA were regarded false positives, whereas sample pairs with mutations found in FFPE tDNA but not PLA ctDNA were regarded as false negatives. Sensitivity, specificity, concordance rate, and plasma predictive value were calculated using a previously published method (27). The concordance rate of gene mutations in tDNA and paired ctDNA was analyzed using Chi-squared test or Fisher exact test in the included NSCLC patients. Statistical analysis was conducted using SPSS 19.0 software (IBM Corporation, Armonk, NY, USA).

## Results

### Study design and physiologic and clinical characteristics of NSCLC cohort

A schematic figure of the study design is illustrated in **Figure 1**. The study was initiated with the collection of FFPE tDNA and/or PLA ctDNA samples from 285 patients (NSCLC alone = 214, NSCLC coexisting COPD = 20, NSCLC coexisting with prodromal changes in COPD = 51). Clinical information including sex, age, BMI, smoking history, cancer type, UICC stage, family history of NSCLC, presenting symptoms and signs, and comorbidities, was collected for 285 patients with NSCLC.

The demographics and clinical characteristics of the NSCLC cohort are depicted in **Table 1**. Relative to the NSCLC alone group, the NSCLC coexisting COPD group and the NSCLC coexisting with prodromal changes in COPD group were characterized by a lower proportion of women, more former and current smokers, lower FEV_1_%, lower proportion of adenocarcinoma patients, in addition to more clinical symptoms, signs, and comorbidity. Moreover, the patients in the NSCLC coexisting with COPD group presented with more symptoms and signs than those in the NSCLC coexisting with prodromal changes in COPD group.

**Table 1:** Patient demographics and clinical characteristics of NSCLC cohort (N=285).

Characteristics	NSCLC alone (N=214)	NSCLC coexisting COPD (N=20)	NSCLC coexisting with prodromal changes in COPD (N=51)
Age (years)	57.53 ± 10.46	66.30 ± 7.21*	64.96 ± 8.87*
Sex (female)	123 (57.48%)	0 (0.00%)*	5 (9.80%)*
Body mass index	22.05 ± 3.78	20.65 ± 6.19	22.45 ± 2.90
Smoking status		*	*
Never(0)	158 (73.83%)	1 (5.00%)	12 (23.53%)
Former(2)	18 (8.41%)	7 (35.00%)	12 (23.53%)
Current(1)	38 (17.76%)	12 (60.00%)	27 (52.94%)
Lung function		*	
FEV_1_/FVC(%)	82.16 ± 6.54	58.35 ± 8.78	72.50 ± 3.62
FEV_1_/predicted(%)	88.03 ± 10.19	69.26 ± 12.50	82.978 ± 9.70
Cell type		*	*
Adenocarcinoma	181 (84.58%)	10 (50.00%)	35 (68.63%)
Squamous cell	9 (4.21%)	6 (30.00%)	12 (23.53%)
Other NSCLC	24 (11.21%)	4 (20.00%)	4 (7.84%)
UICC stage			
Stage I-II	35 (16.36%)	2 (10.00%)	4 (7.84%)
Stage III–IV	145 (67.76%)	15 (75.00%)	38 (74.51%)
Family history of NSCLC	3 (1.40%)	0 (0.00%)	2 (3.92%)
Presenting symptoms and signs			
Cough	135 (63.08%)	17 (85.00%)	40 (78.43%)*
Sputum	98 (45.79%)	15 (75.00%)*	31 (60.78%)
Hemoptysis	37 (17.29%)	8 (40.00%)*#	9 (17.65%)
Dyspnea	52 (24.30%)	11 (55.00%)*†	16 (31.37%)
Chest pain	51 (23.83%)	5 (25.00%)	13 (25.49%)
Fatigue	19 (8.88%)	2 (10.00%)	6 (11.76%)
Dysphagia	1 (0.47%)	2 (10.00%)*†	0 (0.00%)
Hoarseness	4 (1.87%)	5 (25.00%)*#	3 (5.88%)
Pleural effusion	66 (30.84%)	12 (60.00%)*†	20 (39.22%)
Comorbidity			
Pneumonia	55 (25.70%)	10 (50.00%)*	26 (50.98%)*
Coronary heart disease	15 (7.01%)	6 (30.00%)*	8 (15.69%)
Hypertension	55 (25.70%)	9 (45.00%)	14 (27.45%)
Hypercholesterolemia	12 (5.61%)	1 (5.00%)	1 (1.96%)
Diabetes mellitus	21 (9.81%)	4 (20.00%)	5 (9.80%)

*p < 0.05, comparison with NSCLC alone; #p < 0.05 and †p < 0.1, comparison between NSCLC coexisting COPD and NSCLC coexisting with prodromal changes in COPD; Values are mean ± SD or n (%).

### Landscape of gene mutation in NSCLC cohort with FFPE tDNA samples

**Figure 2:**
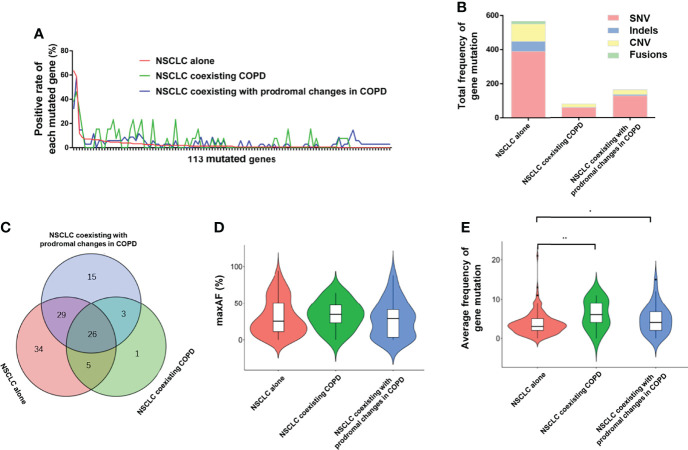
Comparison of FFPE tDNA mutations between the NSCLC alone, NSCLC coexisting with COPD and NSCLC coexisting with prodromal changes in COPD groups. **(A)** The positive rate of each mutated gene in the 113 mutated genes. **(B)** Total frequency of gene mutation and the proportion of each mutated type. **(C)** Venn diagram of mutated genes among the three groups. **(D)** A violin plot of the average maxAF. **(E)** A violin plot of the average frequency of gene mutation.

Furthermore, we carried out capture-based ultra-deep targeted sequencing on all collected FFPE tDNA and PLA ctDNA samples using a panel consisting of 168 genes spanning 170 KB of the human genome. Subsequently, we identified 810 aberrations spanning 113 genes in the NSCLC cohort with FFPE tDNA samples. Meanwhile, no mutations were detected in 5.5% of the patients (11/200). Among the three groups, EGFR, TP53, and KRAS were the most frequently mutated genes (**Figure 2A**), while SNV and CNV were the common genetic aberrations (**Figure 2B**). However, the three groups showed incompletely consistent gene mutation profiles (**Figures 2A, C**). More specifically, we identified a total of 566 aberrations spanning 94 genes in the NSCLC alone group, 80 aberrations spanning 35 genes in the NSCLC coexisting COPD group, and 164 aberrations spanning 73 genes in the NSCLC coexisting with prodromal changes in COPD group (**Table 2**). 31 identical mutated genes existed in the NSCLC alone group (32.98%) and the NSCLC coexisting COPD group (88.57%), 55 identical mutated genes in the NSCLC alone group (58.51%) and the NSCLC coexisting with prodromal changes in COPD group (75.34%), 29 identical mutated genes in the NSCLC coexisting COPD group (82.86%) and the NSCLC coexisting with prodromal changes in COPD group (39.73%) (**Figure 2C**). In addition, the results of gene mutation analysis revealed no statistical difference between the three groups in regard to the positive rate of gene mutation and maxAF (**Table 2** and **Figure 2D**). However, patients in the NSCLC coexisting COPD group and the NSCLC coexisting with prodromal changes in COPD group presented with a higher average frequency of gene mutation compared to patients in the NSCLC alone group (**Figure 2E**).

**Table 2:** Gene mutation profiles of the NSCLC sub-cohort with FFPE tDNA samples (N=200).

Characteristics	NSCLC alone (N=153)	NSCLC coexisting COPD (N=13)	NSCLC coexisting with prodromal changes in COPD (N=34)
Positive rate of gene mutation	146 (92.31%)	12 (95.42%)	31 (91.18%)
Average maxAF (%)	31.24 ± 25.01	33.74 ± 19.92	28.48 ± 24.89
Mutated genes in 168 genes	94	35	73
Total frequency of gene mutation	566	80	164
SNV	389	59	128
Indels	59	2	7
CNV	102	19	27
Fusions	16	0	2
Average frequency of gene mutation	3.70 ± 2.81	6.15 ± 3.63*	4.82 ± 3.55*
Positive rate of COPD dominant genes	17 (11.11%)	8 (61.54%)*#	9 (26.47%)*

*p < 0.05, compared with NSCLC alone; #p < 0.05, comparison between NSCLC coexisting COPD and NSCLC coexisting with prodromal changes in COPD; Values are mean ± SD, n or n (%).

To further identify the mutated genes that could specifically distinguish COPD in NSCLC patients, we investigated the unique mutated genes in the NSCLC coexisting COPD group. Compared with the NSCLC alone group, the four mutated genes (LRP1B, MLH1, EPHA5, and NTRK2) were only present in the NSCLC coexisting COPD group. It is worth noting that three of the aforementioned genes (LRP1B, EPHA5, and NTRK2) were also present in the NSCLC coexisting with prodromal changes in COPD group (**Figure 2C**). Thereafter, we sought to distinguish COPD patients in the NSCLC cohort by searching for combination genes with a higher mutation frequency in the NSCLC coexisting with COPD group.

By assuming the positive rate of the mutated gene as greater than 20% in the NSCLC coexisting COPD group, and less than 20% in the other two groups as the threshold, five genes, namely APC, CSMD3, POLE, FGF3, and CCND1, were identified as COPD dominant genes. As shown in **Table 2**, the positive rate of COPD-dominant genes in the NSCLC coexisting COPD group was significantly higher than that in the other two groups. Subsequently, the NSCLC cohort was divided into two groups according to the presence of mutations in the COPD-dominant genes. As shown in **Table S1**, the higher proportion of smoking patients, the higher proportion of squamous cell carcinoma and the more prone to cough and dyspnea appeared in the positive group of COPD dominant genes (**Table S1**). These results indicate that coexistence of COPD in patients with NSCLC leads to altered gene mutation profiles in FFPE tDNA samples, which may be related to the worse clinical manifestations.

### Landscape of gene mutation in the NSCLC cohort with PLA ctDNA samples

A total of 556 aberrations spanning 111 genes were identified in the NSCLC cohort with PLA ctDNA samples (**Table S2**). Similarly, in the three groups with ctDNA samples, EGFR and TP53 were the most frequently mutated genes (**Figure S1A**), and SNV and CNV were the common aberrations (**Figure S1B**). In addition, all three groups presented with inconsistent gene mutation profiles (**Figure S1A, C**). Meanwhile, patients in the NSCLC alone group still showed more mutated genes in 168 genes and a higher total frequency of gene mutations (**Table S2**). It is also noteworthy that, besides the positive rate of gene mutation and maxAF, there was no statistical difference in the average frequency of gene mutations among the three groups, which was inconsistent with the results uncovered in the FFPE tDNA samples.

Therefore, we identified the inconsistencies in gene mutations between the NSCLC cohort with tDNA samples and the NSCLC cohort with ctDNA samples. Relative to the NSCLC cohort of tDNA samples, the NSCLC cohort of ctDNA samples exhibited a lower positive rate of gene mutation, average maxAF, mutated genes in 168 genes, total frequency of gene mutation, average frequency of gene mutation, and positive rate of COPD dominant genes in the NSCLC alone group, the NSCLC coexisting COPD group, or the NSCLC coexisting with prodromal changes in COPD group (**Figure S2**, **Table 2**, and **Table S2**).

### Mutation concordance in matched FFPE tDNA and PLA ctDNA sample pairs

To further elucidate the mutation concordance in tDNA and ctDNA samples, we screened a total of 110 NSCLC patients with tDNA and paired ctDNA samples. First, we compared the matching degree of mutations between tDNA samples and paired ctDNA samples in the entire cohort. Of the 110 matched tDNA and ctDNA sample pairs, 10 sample pairs presented with completely consistent tDNA and ctDNA mutations, 47 sample pairs presented with partially consistent mutations, and 15 sample pairs presented with completely inconsistent mutations. In addition, 7 sample pairs presented with only ctDNA mutations and 31 sample pairs presented with only tDNA mutations. Overall, the concordance rate of mutations identified in tDNA and paired ctDNA samples was calculated to be 65.45%, with a sensitivity of 64.77% (95% CI 53.79 – 74.45%) and a specificity of 68.18% (95% CI 45.11 – 85.26%), and a PPV of cancer of 89.06% (95% CI 78.16 – 95.12%) (**Table 3**).

**Table 3:** Concordance, specificity, sensitivity and positive predictive value calculations for FFPE tDNA and paired PLA ctDNA samples in the NSCLC sub-cohort (N=110).

	Plasma mutation status			
	Positive		Negative	Total
Tumor mutation status				
Positive	57		31	88
Negative	7		15	22
Total	64		46	110
	**n**	**Rate (%)**	**95% Confidence interval (%)**	
Concordance	110	65.45		
Sensitivity	88	64.77	53.79	74.45
Specificity	22	68.18	45.11	85.26
Positive-predictive value	64	89.06	78.16	95.12

**Figure 3:**
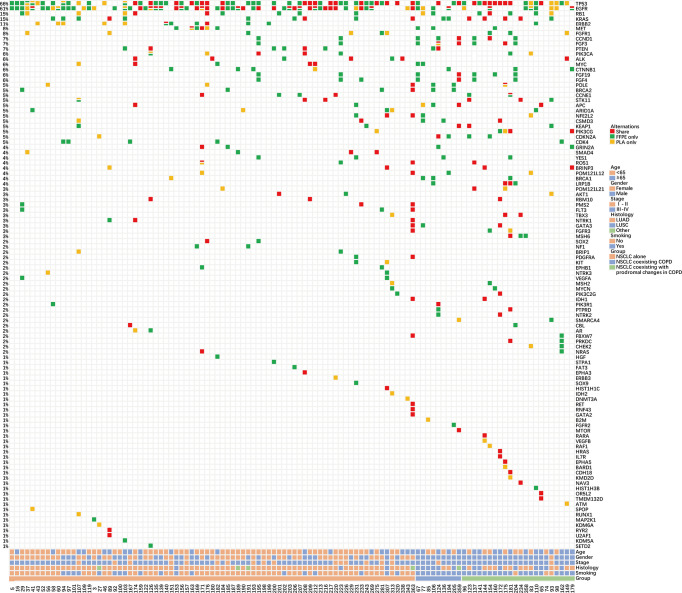
Summary of NSCLC patient characteristics and gene mutations in the matched FFPE tDNA and PLA ctDNA sample pairs. Different colors denote different degrees of gene mutation consistency. The percentage on the side represents the percentage of patients carrying a certain mutation. Bottom bars provide information regarding the age, sex, stage, histological diagnosis, smoking history, and group.

Subsequently, the matching degree of mutations was further evaluated in the three groups with tDNA and paired ctDNA samples (NSCLC alone = 79, NSCLC coexisting COPD = 9, NSCLC coexisting with prodromal changes in COPD = 22) (**Figure 3**). Relative to the whole cohort, similar concordance rate, sensitivity, specificity, and PPV of cancer were noted in the NSCLC alone group and the NSCLC coexisting with prodromal changes in COPD group (**Tables S3** and **S4**). Meanwhile, the concordance rate, sensitivity, specificity, and PPV of cancer in the NSCLC coexisting COPD group were all significantly lower than those in the whole cohort, the NSCLC alone group, and the NSCLC coexisting with prodromal changes in COPD group (**Table S5**). These findings highlight the potential inapplicability of PLA tDNA samples for detecting gene mutations in NSCLC patients with COPD.

Furthermore, we adopted another method to evaluate the degree of matching of mutations in the three groups. Consistent with above results, compared to the NSCLC alone group (41.37%) and the NSCLC coexisting with prodromal changes in COPD group (47.79%), the NSCLC coexisting COPD group exhibited a lower concordance rate of gene mutations (26.67%). The frequency of SNV mutations was the highest among the FFPE tDNA or PLA ctDNA samples in the three groups. However, the concordance rate of fusion mutations was the highest in the NSCLC alone group (75.00%) and the NSCLC coexisting with prodromal changes in COPD group (100%). Meanwhile, there were no fusion and indel mutations, but only SNV and CNV mutations in the NSCLC coexisting COPD group (**Table 4**). Similarly, the overall analysis of eight driver genes mutations (including EGFR, KRAS, ALK, ROS1, MET, RET, HER-2 and BRAF) demonstrated that the NSCLC coexisting COPD group exhibited a lower concordance rate of eight driver genes mutations (7.69%) compared to the NSCLC alone group (45.54%) and the NSCLC coexisting with prodromal changes in COPD group (47.37%) (**Table S6**). These results further prove that the use of ctDNA instead of tDNA may not be the most efficient approach to identifying gene mutations for NSCLC patients with COPD.

**Table 4:** Comparison of genetic mutation profiles for FFPE tDNA and paired PLA ctDNA samples in the NSCLC sub-cohort (N=110).

	Mutation type	Total frequency of gene mutation		Frequency of gene mutation			Concordance rate (%)
		FFPE	PLA	Both in FFPE and PLA	Only in FFPE	Only in PLA	
NSCLC alone (N=79)	Total	278	182	115	163	67	41.37
	SNV	196	138	89	107	49	45.41
	Indels	24	17	11	13	6	45.83
	CNV	50	19	9	41	10	18.00
	Fusions	8	8	6	2	2	75.00
NSCLC coexisting COPD (N=9)	Total	60	23	16	44	7	26.67*#
	SNV	50	19	12	38	7	24.00*#
	Indels	0	0	0	0	0	0.00
	CNV	10	4	4	6	0	40.00#
	Fusions	0	0	0	0	0	0.00
NSCLC coexisting with prodromal changes in COPD (N=22)	Total	113	80	54	59	26	47.79
	SNV	86	77	52	34	25	60.47*
	Indels	4	2	1	3	1	25.00
	CNV	22	0	0	22	0	0.00*
	Fusions	1	1	1	0	0	100.00

*p < 0.05, comparison with NSCLC alone; #p < 0.05, comparison between NSCLC coexisting COPD and NSCLC coexisting with prodromal changes in COPD.

## Discussion

Herein, the current study sought to explore the impact of COPD on the clinical characteristics, gene mutation profiles of 168 NSCLC-related genes, and mutation consistency in tDNA and paired ctDNA of NSCLC patients, and to investigate the impact of these three prodromal changes in COPD on these aspects of NSCLC patients. Our findings revealed that NSCLC patients with COPD exhibited more severe clinical manifestations, compared those with NSCLC alone or NSCLC coexisting with prodromal changes in COPD. In addition, we uncovered that FFPE tDNA samples from the NSCLC coexisting COPD group presented with different gene mutation profiles, including a higher average frequency of gene mutations. Furthermore, our findings identified the decreased concordance between tDNA and paired ctDNA mutations in NSCLC patients with COPD.

As shown by the hard-done work of our peers, our findings highlighted that the coexistence of COPD was associated with worse lung function and more clinical symptoms, signs, and comorbidities in NSCLC patients (9–11). Of note, patients in the NSCLC coexisting COPD group comprised of older men who had a higher proportion of smoking history and squamous cell carcinoma. In accordance with our and other previous studies, male predominance in the NSCLC coexisting COPD group was previously attributed to the male prevalence of cigarette smoking in China (28, 29). In addition, there is a plethora of evidence to suggest that lung squamous cell carcinoma is predominantly found in males, and most profoundly associated with smoking in a dose-dependent manner (30, 31), which is a potential explanation for the phenomenon uncovered in our cohort.

Furthermore, existing evidence indicates that genetic alterations play an important role in the impact of COPD on LC (9, 12). Herein, we consequently speculated that NSCLC patients with COPD may present with different gene mutation profiles from other patients with NSCLC. Subsequent experimentation revealed that in the FFPE tDNA samples of the three groups, the three genes with the highest mutation rate were EGFR, TP53, and KRAS; however, the three groups not only showed differences in the positive mutation rate of the same genes, but also presented with different mutated genes. More specifically, four gene mutations (LRP1B, MLH1, EPHA5, and NTRK2) were only present in the NSCLC coexisting COPD group, which was not true for the NSCLC alone group. Interestingly, all four aforementioned genes have been previously confirmed to be related to LC to varying degrees (32–35), and the LPR1B gene was further highlighted as a predictive biomarker for distinguishing LUAD patients in the presence and absence of COPD (33). Meanwhile, three gene mutations (LRP1B, EPHA5, and NTRK2) were also present in NSCLC coexisting with prodromal changes in COPD, whereas the mutation of MLH1 was only noted in one NSCLC patient with COPD, which indicated that these four genes may not solely be related to COPD. Accordingly, we speculated that some genes that were present at a higher mutation frequency only in the NSCLC coexisting COPD group may be associated with more severe clinical manifestations. We identified five COPD dominant genes (APC, CSMD3, POLE, FGF3, and CCND1), and further validated that mutations in these five genes led to more severe clinical manifestations in NSCLC patients. Unsurprisingly, these five genes have been previously confirmed to be associated with LC to varying extents. For instance, APC mutations are infrequent but present in human LC, such that a prior study indicated the use of promoter methylation of APC as a prognostic marker in NSCLC (36, 37). Meanwhile, the consumption of CSMD3 is known to augment the survival of squamous cell lung cancer tumor cells (38). On the other hand, there is evidence to suggest that POLE mutation represents a candidate biomarker for the response to immunotherapy in NSCLC patients (39). Similarly, co-overexpression of FGF3 and EGFR was previously shown to play critical roles in the pathogenesis of LC (40). CCND1 is closely related to the susceptibility, malignant transformation, treatment effect and prognosis of NSCLC (41–44). Of note, the five genes are less studied with COPD and need further research.

Additional experimentation in our study revealed the presence of fewer mutated genes and total mutation frequencies in the NSCLC coexisting COPD group. As these findings could be attributed to the relatively small sample size of the group, we undertook a different method to tackle this limitation. To exclude the influence of our limited sample size, we further evaluated the average mutation frequency in each group. Interestingly, our findings revealed that compared with the other two groups, NSCLC patients with COPD exhibited a higher average mutation frequency. Altogether, these findings and evidence indicate that the worse clinical manifestations in NSCLC patients with COPD may be associated with the mutations in specific genes.

ctDNA in peripheral blood is commonly known as a “liquid biopsy,” and further serves as a valuable tool in LC patients with tumors that are difficult to biopsy or remove (21, 27). In addition, ctDNA is regarded as an effective replacement for detecting tDNA mutations. However, our findings demonstrated that NSCLC patients with COPD presented with a relatively high average mutation frequency in FFPE tDNA samples, while the same was not true for PLA ctDNA samples. This discovery prompted us to consider the rationality of applying ctDNA to detect gene mutations in NSCLS patients with COPD. Subsequent results of the mutation concordance in tDNA and paired ctDNA samples illustrated that, whether compared with the whole NSCLC cohort, the NSCLC alone group, or with the NSCLC coexisting with prodromal changes in COPD group, there was a significant reduction of the mutation concordance in the NSCLC coexisting COPD group. The latter finding was further validated by a comparison of mutation types dominated by SNV. Many studies have confirmed that no obvious correlation between pulmonary and blood biomarkers in COPD patients, indicating that blood and lung are independent compartments in COPD (45, 46), opposing that the systemic manifestations of COPD are due to the release of lung inflammatory mediators into the circulation (47), which needs further study. This should be responsible for the low concordance rate of gene mutations between tDNA and ctDNA in NSCLC coexisting with COPD group. Collectively, these findings suggest that it may not be suitable to use ctDNA instead of tDNA to detect mutations and monitor treatment response and disease progression in NSCLC patients with COPD.

To the best of our knowledge, our study is the first-of-its-kind to evaluate the impact of COPD on 168 NSCLC-related gene mutation profiles in NSCLC patients. Moreover, our study also possesses the novelty of investigating the rationality of using ctDNA to detect gene mutations in NSCLC patients with COPD. In addition, one of our findings includes the establishment of a method for discovering the impact of gene mutations on clinical manifestations through the combination of COPD-dominant mutated genes. However, we acknowledge three additional limitations that need to be addressed in future studies: first, the study was a retrospective cross-sectional study and lacked “COPD alone group”, which limited our understanding of the development of COPD in LC. Second, NSCLC samples included in our study were collected from a single research center, and the sample size of the NSCLC coexisting COPD group was relatively small. Third, some NSCLC patients with COPD were only diagnosed before surgery, which may influence some of our results. In lieu of the limitations of our study, we emphasize that our findings are indeed preliminary. A larger cohort and a more rigorous design are warranted in the future to validate our findings.

## Conclusion

In summary, our study highlighted that the coexistence of COPD led to worse clinical manifestations and altered gene mutation profiles in patients with NSCLC. Moreover, the coexistence of COPD with NSCLC and its association with worse clinical manifestations could be attributed to certain mutations in specific genes. In addition, the application of ctDNA is not ideal for evaluating gene mutations in NSCLC patients with COPD, which may provide novel guidance for improving the diagnosis, treatment, and efficacy evaluation of NSCLC patients with COPD.

## Data availability statement

The datasets are available in the National Genomics Data Center (NGDC) of China National Center for Bioinformation with accession number HRA003149. This data can be found here: https://ngdc.cncb.ac.cn/gsa-human/browse/HRA003149. Further inquiries can be directed to the corresponding author.

## Ethics statement

The study was approved by the Xiangya Hospital Ethics Review Committee (No. 202204086). Written informed consent for participation was not required for this study in accordance with the national legislation and the institutional requirements.

## Author contributions

Conception and design: LQ, CL, and LY. Administrative support: CH, WY, LQ, and CL. Provision of study materials or patients: LY, CH, WY, and LQ. Collection and assembly of data: LY, TG, WY, XT, and HC. Data analysis and interpretation: CH, YX, XQ, HL, XQ, LQ, and CL. Manuscript writing: All authors. All authors contributed to the article and approved the submitted version.

## Funding

This work was supported by grants #82070034, #81970033, #31900424 from the NSFC; grant #2020SK5361 from the innovation guidance project of clinical medical technology in Hunan province, grant #2021JJ31090 from the project of natural science foundation in Hunan province.

## Acknowledgments

We appreciate the Key laboratory of Basic and Clinical Respiratory Diseases in Hunan Province for offering the equipment and experiment condition.

## Conflict of interest

The authors declare that the research was conducted in the absence of any commercial or financial relationships that could be construed as a potential conflict of interest.

## Publisher’s note

All claims expressed in this article are solely those of the authors and do not necessarily represent those of their affiliated organizations, or those of the publisher, the editors and the reviewers. Any product that may be evaluated in this article, or claim that may be made by its manufacturer, is not guaranteed or endorsed by the publisher.

## Supplementary material

Supplementary Figure 1 Comparison of PLA ctDNA mutations between the NSCLC alone, NSCLC coexisting with COPD and NSCLC coexisting with prodromal changes in COPD groups. (A) The positive rate of each mutated gene in the 111 mutated genes. (B) Total frequency of gene mutation and the proportion of each mutated type. (C) Venn diagram of mutated genes among the three groups. (D) A violin plot of the average maxAF. (E) A violin plot of the average frequency of gene mutation.

Supplementary Figure 2 The difference between FFPE tDNA and PLA ctDNA mutations. (A) A violin plot of average maxAF. (B) A violin plot of the average frequency of gene mutation.
