# Supplementary figures and images for: Clinical characteristics and gene mutation profiles of chronic obstructive pulmonary disease in non-small cell lung cancer
Source: Front Oncol. 2022 Oct 4;12:946881. doi: 10.3389/fonc.2022.946881 (PMC9576924; doi:10.3389/fonc.2022.946881)

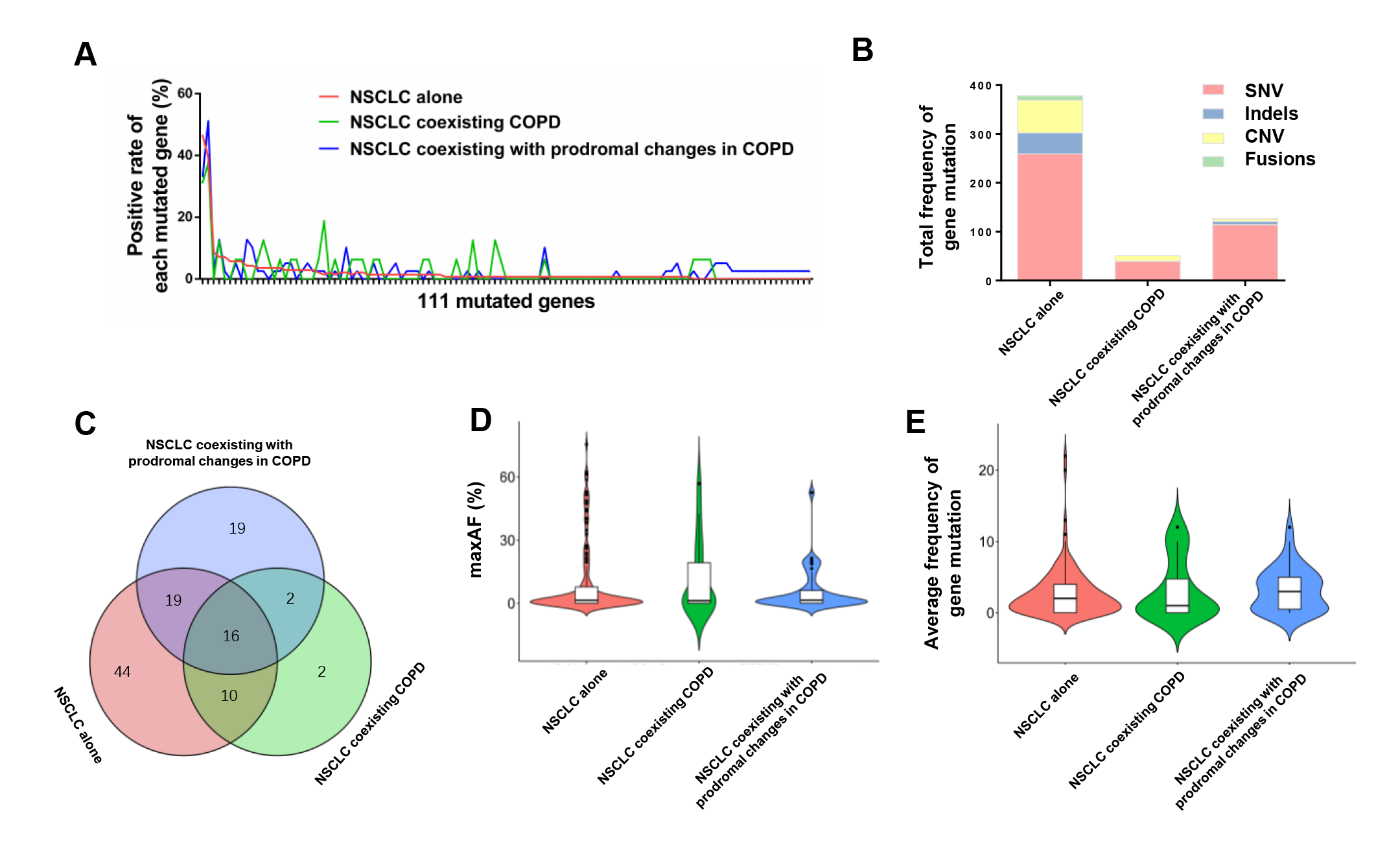

Supplement: Supplementary Figure 1 — Comparison of PLA ctDNA mutations between the NSCLC alone, NSCLC coexisting with COPD and NSCLC coexisting with prodromal changes in COPD groups. (A) The positive rate of each mutated gene in the 111 mutated genes. (B) Total frequency of gene mutation and the proportion of each mutated type. (C) Venn diagram of mutated genes among the three groups. (D) A violin plot of the average maxAF. (E) A violin plot of the average frequency of gene mutation. [file Image_1.tif]

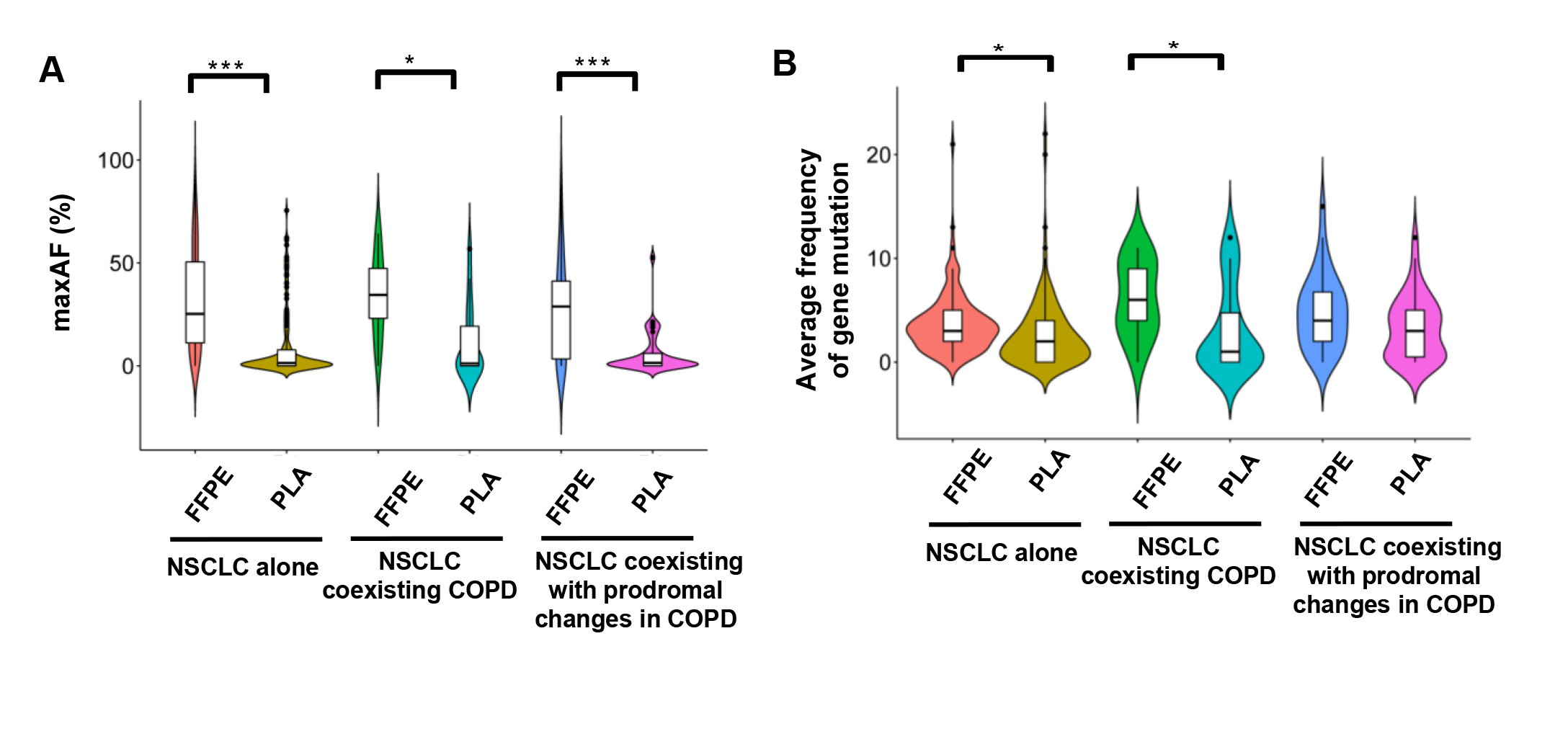

Supplement: Supplementary Figure 2 — The difference between FFPE tDNA and PLA ctDNA mutations. (A) A violin plot of average maxAF. (B) A violin plot of the average frequency of gene mutation. [file Image_2.tif]
