# Supplementary material for: Clinical characteristics and gene mutation profiles of chronic obstructive pulmonary disease in non-small cell lung cancer
Source: Front Oncol. 2022 Oct 4;12:946881. doi: 10.3389/fonc.2022.946881 (PMC9576924; doi:10.3389/fonc.2022.946881)
Supplement: Supplementary file 3 [file Table_1.doc]

**Table S1：Patient demographics and clinical characteristics in the NSCLC sub-cohort with FFPE tDNA samples (N=200).**

| **Characteristics** | **Negative group of COPD dominant genes**  **(N=165)** | **Positive group of COPD dominant genes**  **(N=35)** |
| --- | --- | --- |
| Age (years) | 58.62±10.23 | 61.94±9.63† |
| Sex (female) | 85 (51.51%) | 9 (25.71%)***** |
| Body mass index | 22.23±4.02 | 21.15±4.75 |
| Smoking status |  | ***** |
| Never(0) | 112 (67.88%) | 15 (42.86%) |
| Former(2) | 34 (20.61%) | 12 (34.29%) |
| Current(1) | 19 (11.51%) | 8 (22.86%) |
| Cell type |  | ***** |
| Adenocarcinoma | 141 (85.45%) | 17 (48.57%) |
| Squamous cell | 11 (6.67%) | 11 (31.43%) |
| Other NSCLC | 2 (1.21%) | 3 (8.57%) |
| UICC stage |  |  |
| Stage I-II | 36 (21.82%) | 4 (11.43%) |
| Stage III–IV | 108 (65.45%) | 28 (80.00%) |
| Family history of NSCLC | 3 (1.82%) | 1 (2.86%) |
| Presenting symptoms and signs | | |
| Cough | 105 (63.64%) | 29 (82.86%)***** |
| Sputum | 85 (51.52%) | 22 (62.86%) |
| Hemoptysis | 35 (21.21%) | 6 (17.14%) |
| Dyspnea | 39 (23.64%) | 13 (37.14%)† |
| Chest pain | 39 (23.64%) | 7 (20.00%) |
| Fatigue | 18 (10.91%) | 1 (2.86%) |
| Dysphagia | 2 (1.21%) | 0 (0.00%) |
| Hoarseness | 5 (3.03%) | 3 (8.57%) |
| Pleural effusion | 46 (27.88%) | 13 (37.14%) |
| Comorbidity |  |  |
| Pneumonia | 49 (29.70%) | 14 (40.00%) |
| Coronary heart disease | 13 (7.88%) | 2 (5.71%) |
| Hypertension | 44 (26.67%) | 6 (17.14%) |
| Hypercholesterolemia | 7 (4.24%) | 1 (2.86%) |
| Diabetes mellitus | 17 (10.30%) | 2 (5.71%) |

**p* < 0.05 and †*p* < 0.1, compared with Negative group of COPD dominant genes; Values are mean ± SD or n (%).
