# Supplementary material for: Clinical characteristics and gene mutation profiles of chronic obstructive pulmonary disease in non-small cell lung cancer
Source: Front Oncol. 2022 Oct 4;12:946881. doi: 10.3389/fonc.2022.946881 (PMC9576924; doi:10.3389/fonc.2022.946881)
Supplement: Supplementary file 4 [file Table_2.doc]

# Table S2：Gene mutation profiles of the NSCLC sub-cohort with PLA ctDNA samples (N=195).

| **Characteristics** | **NSCLC alone**  **(N=140)** | **NSCLC coexisting COPD**  **(N=16)** | **NSCLC coexisting with prodromal changes in COPD (N=39)** |
| --- | --- | --- | --- |
| **Positive rate of gene mutation** | 103 (73.57%) | 10 (62.50%) | 29 (74.36%) |
| **Average maxAF (%)** | 9.14±16.50 | 11.22±17.98 | 5.86±10.14 |
| **Mutated genes in 168 genes** | 89 | 30 | 56 |
| **Total frequency of gene mutation** | 378 | 51 | 127 |
| SNV | 259 | 39 | 114 |
| Indels | 43 | 1 | 7 |
| CNV | 67 | 11 | 5 |
| Fusions | 9 | 0 | 1 |
| **Average frequency of gene site mutation** | 2.70±3.34 | 3.19±4.21 | 3.26±3.04 |
| **Positive rate of COPD dominant genes** | 14 (10.00%) | 3 (18.75%) | 6 (15.38%) |

**p* < 0.05, compared with NSCLC alone; #*p* < 0.05, comparison between NSCLC coexisting COPD and NSCLC coexisting with prodromal changes in COPD; Values are mean ± SD, n or n (%).
