# Supplementary material for: Clinical characteristics and gene mutation profiles of chronic obstructive pulmonary disease in non-small cell lung cancer
Source: Front Oncol. 2022 Oct 4;12:946881. doi: 10.3389/fonc.2022.946881 (PMC9576924; doi:10.3389/fonc.2022.946881)
Supplement: Supplementary file 5 [file Table_3.docx]

**Table S3: Concordance, specificity, sensitivity and positive predictive value calculations** **for FFPE tDNA and paired PLA ctDNA samples in NSCLC alone group (N=79).**

|  | **Plasma mutation status** | | |
| --- | --- | --- | --- |
|  | Positive | Negative | Total |
| **Tumor mutation status** |  | | |
| Positive | 41 | 22 | 63 |
| Negative | 5 | 11 | 16 |
| Total | 46 | 33 | 79 |

|  | **n** | **Rate (%)** | **95% Confidence interval (%)** | |
| --- | --- | --- | --- | --- |
| Concordance | 79 | 65.82 |  |  |
| Sensitivity | 63 | 65.08 | 52.75 | 75.67 |
| Specificity | 16 | 68.75 | 41.48 | 87.87 |
| Positive-predictive value | 46 | 89.13 | 75.64 | 95.93 |
