# Supplementary material for: Clinical characteristics and gene mutation profiles of chronic obstructive pulmonary disease in non-small cell lung cancer
Source: Front Oncol. 2022 Oct 4;12:946881. doi: 10.3389/fonc.2022.946881 (PMC9576924; doi:10.3389/fonc.2022.946881)
Supplement: Supplementary file 6 [file Table_4.docx]

**Table S4: Concordance, specificity, sensitivity and positive predictive value calculations for FFPE tDNA and paired PLA ctDNA samples in NSCLC coexisting with prodromal changes in COPD group (N=22).**

|  | **Plasma mutation status** | | |
| --- | --- | --- | --- |
|  | Positive | Negative | Total |
| **Tumor mutation status** |  | | |
| Positive | 13 | 5 | 18 |
| Negative | 1 | 3 | 4 |
| Total | 14 | 8 | 22 |

|  | **n** | **Rate (%)** | **95% Confidence interval (%)** | |
| --- | --- | --- | --- | --- |
| Concordance | 22 | 72.73 |  |  |
| Sensitivity | 18 | 72.22 | 46.40 | 89.29 |
| Specificity | 4 | 75.00 | 21.94 | 98.68 |
| Positive-predictive value | 14 | 92.86 | 64.17 | 99.63 |
