# Supplementary material for: Clinical characteristics and gene mutation profiles of chronic obstructive pulmonary disease in non-small cell lung cancer
Source: Front Oncol. 2022 Oct 4;12:946881. doi: 10.3389/fonc.2022.946881 (PMC9576924; doi:10.3389/fonc.2022.946881)
Supplement: Supplementary file 8 [file Table_6.docx]

**Table S6：Comparison of eight driver genes’ genetic mutation profiles for FFPE tDNA and paired PLA ctDNA samples in the NSCLC sub-cohort (N=110).**

|  | **Mutation type** | **Total frequency of gene mutation** | | **Frequency of gene mutation** | | | **Concordance rate (%)** |
| --- | --- | --- | --- | --- | --- | --- | --- |
|  |  | **FFPE** | **PLA** | **Both in FFPE and PLA** | **Only**  **in FFPE** | **Only**  **in PLA** |  |
| **NSCLC alone**  **(N=79)** | **Total** | 101 | 64 | 46 | 55 | 18 | 45.54 |
|  | SNV | 50 | 39 | 26 | 24 | 13 | 52.00 |
|  | Indels | 20 | 12 | 9 | 11 | 3 | 45.00 |
|  | CNV | 23 | 6 | 5 | 18 | 1 | 21.74 |
|  | Fusions | 8 | 7 | 6 | 2 | 1 | 75.00 |
| **NSCLC coexisting COPD**  **(N=9)** | **Total** | 13 | 3 | 1 | 12 | 2 | 7.69*# |
|  | SNV | 7 | 3 | 1 | 6 | 2 | 14.29 |
|  | Indels | 1 | 0 | 0 | 1 | 0 | 0.00 |
|  | CNV | 5 | 0 | 0 | 5 | 0 | 0.00 |
|  | Fusions | 0 | 0 | 0 | 0 | 0 | 0.00 |
| **NSCLC coexisting with prodromal changes in COPD**  **(N=22)** | **Total** | 19 | 15 | 9 | 10 | 6 | 47.37 |
|  | SNV | 12 | 13 | 7 | 5 | 6 | 58.33 |
|  | Indels | 4 | 1 | 1 | 3 | 0 | 25.00 |
|  | CNV | 2 | 0 | 0 | 2 | 0 | 0.00 |
|  | Fusions | 1 | 1 | 1 | 0 | 0 | 100.00 |

**p* < 0.05, comparison with NSCLC alone; #*p* < 0.05, comparison between NSCLC coexisting COPD and NSCLC coexisting with prodromal changes in COPD.
